# Chicago Medical Society, September 21, 1874

**Published:** 1874-10-15

**Authors:** Will. T. Montgomery


### CHICAGO MEDICAL SOCIETY.

### MEETING OF SEPTEMBER 21ST, 1874.

*Reported by Will. T. Montgomery, M. D.*

DR. DANFORTH presented microscopical specimens of two ovarian cysts. The first was from a tumor of long standing, removed about one month ago by Dr. Wm. E. Clark. It was for a time doubtful as to the nature of the tumor. He was requested to examine some of the fluid from it, and found Eichwald's gorged granules and crystals of colesterin. He had never found either of these from any except an ovarian cyst. These granules are believed to be degenerated cells. The granules and crystals of colesterin were both clearly shown in this specimen.

The second specimen was of colloid cells from a tumor removed by Dr. John E. Owens, about six months ago. The tumor in this case was not of so long standing. These cells are found in the early development of ovarian cysts, and the granules in the latter.

Dr. Bridge inquired as to the difference between colloid cells and cancer cells, and if the former were malignant.

Dr. Danforth. The difference is the colloid cells are all of the same type—cancer cells may have any form. He thought if a morbid growth presents cells all of a certain type it is not malignant.

Dr. Steele reported the following case of ovariotomy for Dr. Quine, and presented a specimen of the cyst.

Regina N——, aged 27; unmarried; has menstruated regularly since the 16th year of her age. Five years ago her abdomen began to enlarge, and she noticed also that pain attended the first two days of the menstrual flow. In August, 1872, after severe muscular exercise, she was seized with pain in the right lumbar region, that continued several months, and was accompanied by fever, progressive emaciation, and rapid increase in the size of the abdomen. So burdensome did the enlargement become, that she was incapacitated for work, and was obliged in May, 1873, to seek relief in the county hospital. She was extremely emaciated then, her skin was sallow, and her features were expressive of anxiety and suffering. From May, 1873, to August, of this year, she was tapped six times, the aggregate amount of fluid evacuated being one hundred and sixty pounds. The fluid was of a dark greenish color at first, very viscid and thick, and contained a copious admixture of pus; but its character improved with each succeeding paracentesis, and the accumulation became more rapid. In two instances the operation was followed by peritonitis. From the first tonics were employed, and hygienic measures instituted, with a view to improving nutrition; but ovariotomy was then considered impracticable, because of the supposed existence of pulmonary tubercle. The condition of the patient steadily improved, and a pleurisy and pericarditis under which she had been laboring got well. A month after the last tapping, the abdomen having again become moderately distended, it was decided, by a consultation between the attending physician and Drs. Byford and Fitch, that operation was practicable. On the 12th of August, Dr. Quine, assisted by Drs. Fitch and Bogue, and the house staff of the hospital, performed the operation, making an incision four and a half inches long in the mesial line and carrying it rapidly through the walls of the abdomen. The trifling hæmorrhage from the wound was readily checked. When the peritoneum was cut and the finger of the operator passed through, he failed to discover any adhesions in the circle of its reach. Thirty pounds of fluid were then evacuated by a large trocar, and as the sac was being withdrawn, adhesions to the omentum and right parieties were revealed. Momentary embarrassment was occasioned by a little aggregation of cysts that resembled a kidney in size, shape and color. The omental adhesions, though not very extensive were exceedingly vascular, and the parietal adhesions were also vascular and very firm. Some parts of the adhering omentum were tied and cut off, and other parts were gently separated from the cyst walls and left.

Some delay was occasioned by persistent bleeding from the parietal adhesions, but sufficient time was allowed to check all oozing. The pedicle was ligated by transfixion and double ligature, and cut short. The abdominal cavity was carefully sponged with tepid salt water, and the wound closed with six silver wire sutures. A compress and bandage completed the dressing. After reaction was fairly established, not a single unfavorable symptom appeared, the wound healing perfectly in six days, and the patient being able to sit up in nine after the operation.

Dr. Gapen reported the following case also for Dr. Quine, and exhibited tumor.

The Doctor saw the patient, a woman, 25 years of age, in a consultation which was necessitated by malposition of the child in parturition. The labor terminated favorably. Subsequently, owing to the removal of the previous attendant from the city, Dr. Quine was recalled. He then learned that the lady had been suffering for seven years from bronchitis and gastro-enteritis, and was reduced to a state of extreme emaciation by these affections. At the first visit the irritability of the stomach was so great that the blandest articles of food were retained with difficulty, and only in very small quantities. Vast quantities of gas were being incessantly eructated and passed from the bowels. The patient was also suffering from harassing cough, with profuse expectoration, and a very distressing tenesmic diarrhœa.

She improved in condition slowly and unsteadily for several months, and finally, at a time when her health had become better than it had been for years, passed temporarily from the doctor's care. In November of 1873, she returned, in consequence of an exceedingly sensitive tumor that occupied the pelvic cavity, and protruded from the vagina when she assumed a standing posture. She was then seven months pregnant, and complained of a return of the old bronchial and gastro-enteric disorder. A cautious digital examination in his office yielding negative results, the Doctor sent the lady home, promising to call the following evening. He did so, finding her well advanced in labor, the shoulder of the child presenting. The presentation was made out with great difficulty, owing to the occupation of the pelvis by a sensitive, fluctuating tumor, and consequent displacement of the uterus, the os pointing to the umbilicus, and being very high. Chloroform was administered, and the child easily delivered, by pedalic version. As the child was being withdrawn, the tumor, which had been no impediment to labor, ascended into the abdominal cavity. The patient made a very tardy recovery, owing to the occurrence of pelvic cellulitis; and before she was able to leave the bed, a diagnosis of ovarian tumor was made. During the succeeding seven months the lady was tapped three times. The second and third tapping yielding respectively less fluid and correspondingly less relief than the preceding. At the third tapping, performed in consultation with Dr. T. D. Fitch, only about a quart of fluid was withdrawn. The abdomen of the patient had reached an enormous size, and the gastro-enteric disorder coupled with constant pelvic distress, contributed to make life miserable, and threateningly short. Ovariotomy was decided upon by Drs. Bartlett, Bogue, Fitch, and Quine, as the only means that offered the slightest prospect of relief or long continuance of life. Accordingly, and notwithstanding the extreme feebleness and emaciation of the patient, and the existence of grave gastro-enteric disease, the operation was performed by Dr. Quine, assisted by Drs. Bogue, T. D. Fitch, Stillians, and myself. The operation was tedious and very difficult. The abdominal incision extended from an inch and a half above the umbilicus to within an inch of the pubes. The tumor was firmly adherent to all parts of the abdominal walls, and an attempt to pass the grooved director between the cyst and the peritoneum resulted in the rupture of the thin and lacerable walls of the tumor, and the escape of its fluid contents. The omental adhesions were extensive, but there was no adhesion to any other abdominal, nor to any pelvic viscus. The great mass of the tumor consisted of colloid matter, which in order to remove through the wound in the abdomen, it was necessary to break down into handfuls. The mass being removed, the pedicle, which was short and only moderately thick, tied by transfixion and double ligature, the abdominal cavity was cleansed with sponges and salt-water, of its colloid and cystic contents, the abdominal wound was closed by six equidistant silver wire sutures. The patient was then put to bed between woolen blankets, a number of hot bricks were applied, and alcoholic stimulants, to promote reaction. The wound was dressed with compress and bandage. Beef tea and brandy were cautiously given per rectum, care being taken to avoid provoking movement of the bowels, by injecting slowly and in small quantities. The catheter was used regularly, at intervals of six hours. After reaction was well established the patient commenced to vomit, and vomited almost incessantly for three or four days. Morphine was given hypodermically, in quantities sufficient to allay pain and procure rest, and quinine was administered in the same way to the amount of about fifteen grains daily. Bismuth in twenty-grain doses and ice allayed the irritability of the stomach sufficiently to enable the patient to take considerable quantities of milk. The convalescence of the patient has been much retarded by the old inflammatory disorders, and by an accumulation of putrescent fluid in the peritoneal cavity, which was limited by pelvic and abdominal adhesions. The pressure of this fluid in the pelvic cavity gave rise to a general cellulitis, and the sufferings of the patient were further increased by the occurrence of cystitis. Ten or twelve days after the operation Dr. Bogue, in consultation with Drs. Fitch and Quine, drew off, through the cul de sac of Douglass, by means of the aspirator, nearly three pints of a grumous fetid fluid, affording prompt and great relief to the pelvic distress. There has been no considerable accumulation of fluid, a spontaneous opening into the intestine affording ready drainage of the cavity. The treatment of the case has, in consequence of the complications, been widely different from the plan ordinarily followed, and combined measures directed to the cure of the old intestinal disorder and the acute inflammation of the bladder. But, notwithstanding these serious complications and alarming drawbacks to convalescence, the patient has been improving steadily but slowly, so that at the present, perfect recovery may be confidently predicted. I am requested by Dr. Quine to gratefully acknowledge his indebtedness to Drs. Bogue and Fitch for frequent and valuable counsels.

Dr. Hutchinson gave a verbal report of two interesting cases of ovariotomy which he had had. He thought his cases were more successful than the others reported; for besides recovering without a bad symptom, each patient became pregnant after the operation.

Dr. S. J. Avery reported a case of Sciatica, of several years' standing, cured in three weeks, by replacing a prolapsed uterus. A full report of which appears elsewhere in the present number of the EXAMINER.

The discussion of this case was on motion postponed until next meeting.

---

PROF. SCHIFF, ON THE DIFFERENCE IN THE ANÆSTHESIA PRODUCED BY CHLOROFORM AND ETHER.—As a result of several thousand experiments on animals, the author has come to the conclusion that ether *gradually* suspends the processes of respiration and circulation, so that it is in the power of the operator to save the animal at any given moment, while the suspension produced by chloroform is not gradual but irregular, the circulation often ceasing while respiration still exists. The author would, therefore decide *that the physician is responsible for any death by ether, but not for a fatal issue produced by chloroform.*

The Florence Academy has deemed this communication of sufficient interest to elect a committee to investigate these statements, and to test some of the means proposed by Dr. Schiff to avoid the dangers of anæsthesia.

